# Visceral adipose tissue: emerging role of gluco- and mineralocorticoid hormones in the setting of cardiometabolic alterations

**DOI:** 10.1111/j.1749-6632.2012.06597.x

**Published:** 2012-07-17

**Authors:** Marco Boscaro, Gilberta Giacchetti, Vanessa Ronconi

**Affiliations:** Division of Endocrinology, Ospedali Riuniti “Umberto I-G.M. Lancisi-G. Salesi,” Università Politecnica delle MarcheAncona, Italy

**Keywords:** adipose tissue, glucocorticoids, mineralocorticoids

## Abstract

Several clinical and experimental lines of evidence have highlighted the detrimental effects of visceral adipose tissue excess on cardiometabolic parameters. Besides, recent findings have shown the effects of gluco-and mineralocorticoid hormones on adipose tissue and have also underscored the interplay existing between such adrenal steroids and their respective receptors in the modulation of adipose tissue biology. While the fundamental role played by glucocorticoids on adipocyte differentiation and storage was already well known, the relevance of the mineralocorticoids in the physiology of the adipose organ is of recent acquisition. The local and systemic renin–angiotensin–aldosterone system (RAAS) acting on adipose tissue seems to contribute to the development of the cardiometabolic phenotype so that its modulation can have deep impact on human health. A better understanding of the pathophysiology of the adipose organ is of crucial importance in order to identify possible therapeutic approaches that can avoid the development of such cardiovascular and metabolic sequelae.

## Introduction

Overweight and obesity have become a major public health problem in industrialized countries. Epidemiological studies have highlighted a rapid increase of such conditions worldwide, with a prevalence of about 30% and 20%, respectively, in the general population of the United States of America.^1^ Along with the increase in obesity there is a parallel increase in the prevalence of obesity-related diseases, such as type 2 diabetes, impaired glucose tolerance,^2^,^3^ and arterial hypertension.

The combination of excessive food intake and reduced physical activity—with consequent overweight/obesity—can lead, in subjects who are genetically predisposed, to insulin resistance in peripheral tissues, such as skeletal muscle, liver, and adipose tissue, which is the *primum movens* for other pathological conditions. Insulin resistance is indeed a pathogenetic element that plays a key role in the development of metabolic and hemodynamic alterations and is responsible, in turn, for the onset of the so-called cardiometabolic syndrome.^4^

But what is the role of adipose tissue in this scenario? Adipose tissue, traditionally regarded simply as an inert energy storage organ, is appreciated increasingly as an endocrine organ and an important part of the innate immune system. In the last few years it has been recognized that adipose tissue can produce and secrete into the blood stream a wide variety of bioactive mediators named *adipokines*. The relevance of adrenal steroid excess, and in particular of cortisol—and more recently of aldosterone—has been proposed, on the basis of old and new clinical and experimental studies, to be involved in the pathogenesis of metabolic complications.

## Adipose tissue

Histologically, two fundamentally different adipose tissue types can be differentiated: white adipose tissue (WAT) and brown adipose tissue (BAT). Additionally, its anatomical distribution further classifies adipose tissue as either subcutaneous or visceral. Rodents have WAT and BAT in distinct depots, epididymal and interscapular, respectively, while the topographic distribution of BAT in humans is slightly different. Humans are born with BAT located mainly around the neck and large blood vessels of the thorax that is then partially replaced by WAT in adults.

Traditionally, the main functions ascribed to adipose tissue have been insulation, mechanical support, and storage of surplus fuel. In fact, in the presence of increased food intake and/or decreased energy expenditure, surplus energy is deposited through the action of lipogenic enzymes in the form of neutral triglycerides in adipocyte droplets. Conversely, when food is scarce and/or energy expenditure requirements increase, lipid reserves are released through lipolitic enzymes to provide fuel (i.e., free fatty acid, FFA) for energy generation in peripheral tissues and organs, as in the liver, muscle, and BAT.

**Figure 1:**
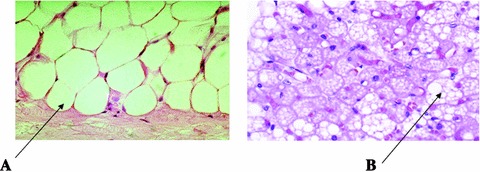
Morphological appearance of white (left) and brown (right) adipose tissue (WAT and BAT). (A) Unilocular adipocyte; (B) multilocular adipocyte.

From a morphological point of view, BAT and WAT are formed by different adipocytes. While white adipocytes form only single large vacuoles (unilocular cells) that contain triglycerides, brown adipocytes form numerous small vacuoles (multilocular cells) as “quick-access” fuel for heat production through mitochondrial uncoupling of oxidative phosphorylation of FFA^5^ (Fig. 1). This thermogenic process is of vital importance in neonates exposed to the cold, and still persists in adult humans, in whom substantial amounts of metabolically active BAT have been detected in the paracervical and supra-clavicular region.^6^ The signal for the activation of BAT via activation of the sympathetic nervous system is a temperature below thermoneutrality (34 °C for mice, 28 °C for rats, and 20–22 °C for humans). A second known stimulus for activation of BAT is food intake, thus suggesting the hypothesis of antiobesity properties of BAT.^7^

Adipose tissue is not a homogenous organ. It consists of a variety of different cell types: adipocytes, preadipocytes, stromal/vascular cells, and macrophages.^8^ Macrophages are known to be crucial contributors to inflammation, and quite recently it has been shown that WAT inflammation, due to macrophage infiltration, is a relevant and early event in the development of obesity-related complications.^9^–^12^ Cinti *et al.* have also shown that macrophage infiltration in both murine and human WAT of obese subjects is linked to adipocyte death.^13^ The majority of such macrophages, likely attracted to phagocyte lipids and cellular debris, surround dead adipocytes, forming crown-like structures.

More recently, it has been recognized that adipocytes also demonstrate intrinsic inflammatory properties, and, like macrophages, they sense the presence of pathogens and inflammation and activate multiple inflammatory signal transduction cascades that result in the secretion of several inflammatory cytokines and acute-phase reactants.^14^

## Adipose tissue as an endocrine organ

In 1994, the discovery of leptin—a satiety factor—as an adipocyte-secreted protein^15^ led to the definition of WAT as an endocrine organ. Systemic analysis of the active genes in WAT, after constructing a 3′-directed complementary DNA library, revealed a high frequency of genes encoding secretory proteins.

**Figure 2:**
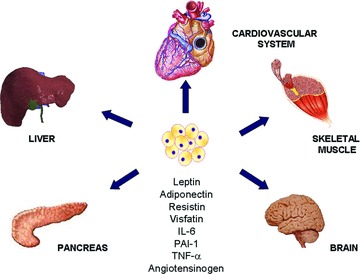
List of some adipokines secreted by the adipose organ and representation of the biological systems affected by such humoral products.

As said, adipose tissue contains different cell types; and, importantly, each of these cell types presents its own secretion profile and specific regulation. The additional cell types present in the adipose tissue, or its stromal-vascular fraction, include pericytes and endothelial cells, monocytes, macrophages, and pluripotent stem cells (including preadipocytes). Interestingly, these nonadipocyte cells may also be the main source of some secreted factors. The adipose organ expresses indeed more than 8,000 genes, including those for more than 120 receptors and 80 secreted proteins and hormones. Approximately 20–30% of all genes in WAT encode secretory proteins.^16^ Such humoral products are involved in processes such as inflammation, lipid metabolism, energy balance, vascular tone, and atherosclerosis, but also glucose homeostasis and insulin sensitivity.^17^ Leptin, adiponectin, plasminogen activator inhibitor (PAI 1), and components of the renin–angiotensin–aldosterone system are only a few of the substances produced by adipose tissue, which can act both with autocrine/paracrine mechanisms and in an endocrine manner—thus adipose tissue can be fully considered an adipose organ (Fig. 2).^18^

The relevance of adipocytes for health was shown through the use of the animal model of lipoatrophic diabetes,^19^ in which mice had virtually no white fat tissue due to genetic manipulation. These animals displayed insulin resistance, hyperglycemia, hyperlipidemia, and fatty livers—all the characteristics of humans with severe lipoatrophic diabetes. This particular phenotype was completely reversed by the transplantation of adipose tissue from healthy mice, highlighting that the absence of adipocytes is metabolically detrimental. Conversely, WAT, especially the visceral fat, which is more hormonally active than the subcutaneous one, can also act as a “bad guy.” It can potentially become one of the largest organs in the body, and when that happens, the total number of adipokines secreted from WAT can affect whole-body homeostasis. The massive increase in fat mass leads in fact to a dysregulation of circulating adipokine levels that may have pathogenic effects associated with obesity by triggering obesity-associated disorders, including systemic inflammation, insulin resistance, hypertension, hyperlipidemia, type 2 diabetes, and coronary heart disease.

In 1988, the American Heart Association identified obesity as a major, modifiable risk factor for coronary heart disease.^20^ In particular, visceral obesity, that is, accumulation of adipose tissue within the abdomen, is associated with cardiovascular and metabolic complications.^21^,^22^ Health problems are indeed more closely correlated to *android* obesity (visceral obesity or upper body obesity) than to *gynoid* obesity (lower body obesity). At a clinical level, visceral obesity is characterized by an increase in the waist-to-hip ratio, and this measurement is better than BMI as an indicator of cardiovascular and metabolic risk.^23^

## Adipose tissue and the cardiometabolic syndrome

Cardiometabolic syndrome refers to the clustering of obesity-related metabolic disorders in one individual; in particular, it defines a syndrome characterized by the presence of glucose and lipid profile alterations, insulin resistance, hypertension, and, finally, cardiovascular diseases. This condition affects one in four adults, making it the leading public health issue associated with increased cardiovascular disease risk in the industrialized world.^24^ The mechanisms that causally relate visceral obesity and metabolic syndrome—and therefore cardiovascular diseases—are not fully understood.

A hallmark of cardiometabolic syndrome is undoubtedly insulin resistance. Insulin resistance is defined as an inadequate response by insulin target tissues, such as skeletal muscle, liver, and adipose tissue, to the physiologic effects of circulating insulin. Impaired insulin sensitivity in these three tissues leads to reduced insulin-mediated glucose uptake by skeletal muscle, impaired insulin-mediated inhibition of endogenous glucose production in the liver, and a reduced ability of insulin to inhibit lipolysis in adipose tissue. Insulin resistance is a major predictor of the development of various metabolic complications, such as type 2 diabetes. It is indeed well established that in type 2 diabetes overt hyperglycemia is preceded by insulin resistance.^25^ The causes of insulin resistance can be genetic and/or acquired, yet the genetic causes or predispositions toward insulin resistance in prediabetic populations are poorly understood. Although inherited defects in the basic insulin signaling cascade have been proposed,^26^ it is likely that any genetic component must interact with environmental factors. In industrialized countries, the most common acquired factors causing insulin resistance are obesity, sedentary lifestyle, and aging—all of which are interrelated.^27^ From a pathogenetic point, many lines of evidence have shown that chronic activation of proinflammatory pathways within insulin target cells can lead to obesity-related insulin resistance. Consistent with this, elevated levels of the proinflammatory cytokines TNF-α, IL-6, and C-reactive protein (CRP) have been shown in individuals with insulin resistance and diabetes.^28^–^30^

Elevated plasma glucose is sensed by pancreatic beta cells, which increase insulin secretion to compensate for hyperglycemia, resulting in circulating hyperinsulinemia. However, over time, beta cells fail to secrete insulin normally and can no longer compensate for the decreased tissue insulin sensitivity, with consequent development of impaired glucose tolerance and eventually type 2 diabetes.^25^ An increase of circulating FFA levels is observed before patients with insulin resistance develop glucose metabolism alterations. Impairment of insulin signaling in adipose tissue leads to increase of lipolysis and, possibly, defective storage of FFA in adipocytes.^31^,^32^ Insulin resistance is also responsible for decreased activity of lipoprotein lipase and limited degradation of apoB. Together, these actions induce hypertriglyceridemia, characteristic of insulin resistance, and low aHDL phenotype, which are implicated in the development of atherosclerosis.^33^

Another relevant aspect of metabolic syndrome is its association with high blood pressure. Several mechanisms have been proposed to explain the pathogenesis of hypertension in relation to obesity and insulin resistance. Some of the physiological and tissue-specific consequences by which insulin resistance could result in hypertension include changes in vascular structure and function, alterations in cation flux, activation of the sympathetic nervous system, and enhanced renal sodium retention.^34^ In this regard, the antinatriuretic action of insulin has been highlighted. The insulin-mediated reduction in Na^+^ excretion appears to be mainly due to increased Na^+^ reabsorption at the level of Henle's loop.^35^,^36^ Chronic hyperinsulinemia can thus cause the rise in blood pressure through an increase in extracellular volume and cardiac output. Insulin can also enhance renal sodium retention through stimulation of the sympathetic nervous system and augmentation of angiotensin II–mediated aldosterone secretion.^37^,^38^

Moreover, the increase in FFA release by adipose tissue, which is observed in insulin resistant states, has a direct effect on peripheral resistance, as well as an effect mediated by the inhibition of NO synthase. In addition, insulin and insulin-like growth factors are mitogens capable of stimulating smooth muscle proliferation.^39^ Therefore, hyperinsulinemia could result in vascular smooth muscle hypertrophy responsible for increased vascular resistance that, ultimately, leads to the development of high blood pressure. Another role for hyperinsulinemia in the etiology of hypertension related to insulin resistance is via upregulation of AT1R; this potentiates the physiologic actions of AngII, which include peripheral vasoconstriction and plasma volume expansion.^40^,^41^

Finally, metabolic syndrome is a condition characterized by proinflammatory and prothrombotic states. Elevation of cytokines (e.g., TNF-α and IL-6), as well as acute-phase reactants (CRP and fibrinogen), is indeed peculiar to the syndrome. Elevated CRP—defined as a risk factor for CVD^42^—seems to be linked to obesity, as excess adipose tissue releases inflammatory cytokines that may lead to higher CRP levels and to higher levels of the prothromotic factors plasminogen activator inhibitor (PAI)-1 and fibrinogen. Thus, prothrombotic and proinflammatory states may be metabolically interconnected.^43^ But what is the interplay between visceral adipose tissue, the cardiometabolic syndrome, and adrenal steroids such as gluco- and mineralocorticoids?

## Adipose tissue and glucocorticoids

Close phenotypic similarities exist between metabolic syndrome and conditions characterized by chronic exposure to glucocorticoid hormones, either exogenous or endogenous, such as in Cushing syndrome (CS). Common features are abdominal obesity, insulin resistance, hypertension, hyperglicemia, and dyslipidemia, which are all comorbidities strictly associated with the presence of obesity itself.

Obesity in CS is characterized by increased food intake with no changes in energy expenditure, reduced lean mass, and increased body weight with a redistribution of fat mass from peripheral toward central sites of the body, mainly in the truncal region and visceral depots.^44^ This is not surprising if one considers that glucocorticoids (GCs) promote both the differentiation and the proliferation of human adipocytes through glucocorticoid receptors (GRs), which are more abundantly expressed in visceral than in subcutaneous adipose tissue.^45^

Although not univocal, several lines of evidence support the role of cortisol in the pathogenesis of obesity. Higher fasting cortisol levels have been found in patients with central obesity;^46^ cortisol levels have been found to correlate with waist circumference,^47^–^48^ while patients with abdominal obesity display an increased responsiveness of the hypothalamic–pituitary–adrenal (HPA) axis to a wide variety of stimuli, including food intake,^49^ a low-dose ACTH test,^50^ and CRH arginine vasopressin test.^51^ In addition, in patients with central obesity, a loss of diurnal cortisol variation as well as a resistance to a low oral dexamethasone suppression test^52^ have been described, suggesting hyperactivity of the HPA axis. Also increased urinary-free cortisol excretion has been found by different authors.^53^–^55^

### Role of the glucocorticoid receptor polymorphisms

In the pathogenesis of GC-induced obesity, the relevance of individual sensitivity to GC exposure has been proposed and supported by genetic studies on the GR.^56^ The GR is a ubiquitously expressed protein, encoded by exons 2–9 of the GR gene, located on chromosome 5 (5q31–32).^57^,^58^ Alternative splicing of the GR precursor mRNA gives rise to 5 GR protein subtypes, termed GRα, GRβ, GRγ, GR-A, and GR-P. GRα is the functionally active protein, made up of 777 amino acids, and differs from GRβ only by its *C*-terminal residues.^59^ Classically, GRβ has been considered a regulator of GRα via a dominant-negative effect. However, it has been recently demonstrated that GRβ also seems to have a role in cell signaling, regulating gene expression even in the absence of GRα.^60^

**Figure 3:**
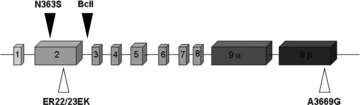
Schematic representation of the glucocorticoid receptor (GR) gene and its polymorphisms: N363S, codon 363 AAT→AGT asparagine (N) to serine (S) substitution; *BCl*1, intron 2 C→G substitution, 646 nucelotides downstream from exon 2; ER22/23EK codon 22 GAG → GAA+ codon 23 AGG → AAG substitution with consequent arginine (R) to lysine (K) substitution. The black arrows indicate polymorphisms that increase the sensitivity to glucocorticoids, while the white arrows refer to polymorphisms that confer resistance to glucocorticoids.

Polymorphisms in GR genes have been associated with variations in GR function^61^,^62^ (Fig. 3). In particular, ER22/23EK carriers have a relative resistance to GCs and display a more favorable metabolic profile, including increased insulin sensitivity, and lower cholesterol levels and cardiovascular risk compared to wild-type carriers.^63^ They also have beneficial effects on body composition (increased lean mass, lower fat mass, and same BMI) compared with noncarriers.^64^

On the other hand, N363S and *Bcl*I variations are associated with hypersensitivity to GCs, and may predispose to obesity.^65^–^67^

Although contrasting data have been reported on the association between *Bcl*I polymorphism and body composition,^68^–^70^ it can be speculated that hypersensitivity to GCs has negative effects on abdominal fat mass early in life, while later in life GCs mainly affect lean mass by lowering it.^63^ To confirm such a hypothesis, a recent study has found an association between BMI, fat mass, and *Bcl*I variation in young carriers, while in older subjects the same variant is associated with lower BMI due to lower lean mass.^71^

As for the N363S variant, although a few studies found no association between it and BMI,^72^,^73^ numerous other observations, conducted in different cohorts of subjects, have highlighted a contribution of the N363S variant to overweight and obesity.^67^,^74^,^75^ Another studied GR polymorphism is the A3669G variant, which has been associated in Caucasian men with reduced central obesity and more favorable lipid profiles.^76^ In support of a protective role by the A3669G polymorphism, a recent study found one in patients with CS for the development of diabetes mellitus.^77^ Interestingly, in a subset of patients affected by eating disorders (anorexia nervosa, bulimia nervosa, and binge eating disorder) and obesity, the A3669G polymorphism was associated with binge eating disorder, while the N363S variant was associated with higher BMI independent of eating psychopathology.^78^

### Role of 11β-hydroxysteroid dehydrogenase type 1

If one looks at GC action variability, one can distinguish between receptor level (i.e., at the GR) and prereceptor level action. Another fundamental variable that can modulate GC action in peripheral tissues is the activity of 11β−hydroxysteroid dehydrogenase type 1 (11β-HSD1), which determines GC availability at the prereceptor level in such tissues.

Circulating cortisol levels are not always increased in obese patients;^79^–^81^ increased metabolic clearance has been claimed as a possible explanation for this,^82^ but GC concentrations at a local level strictly linked to 11β-HSD1 activity is also of major importance. 11β-HSD1 is ubiquitously expressed and interconverts inactive cortisone and 11-dehydrocorticosterone to their active compounds cortisol and corticosterone. 11β-HSD1 is a bidirectional enzyme, although it acts predominantly as a reductase (converting inactive cortisone to active cortisol) rather than as a dehydrogenase, and it is highly expressed in adipose tissue, where it amplifies GC action independent of circulating cortisol levels.^83^ Genetic studies in animal models have shown that 11β-HSD1 expression or activity plays a key role in determining a metabolic syndrome phenotype. Transgenic mice overexpressing 11β-HSD1 selectively in adipose tissue present visceral obesity, exaggerated by a high-fat diet, diabetes, insulin resistance, hyperlipemia, and hyperphagia.^84^ Interestingly, overfed wild-type mice show reduced 11β-HSD1 expression in adipose tissue, indicating regulation of enzyme expression according to energy balance.^84^ Hepatic overexpression of 11β-HSD1 in transgenic mice induces fatty liver, dyslipidemia, hypertension, and insulin resistance without obesity.^85^ Knockout mice for 11β-HSD1 have a reduced risk for obesity and metabolic syndrome,^86^ and data from our group support a tight correlation between obesity and 11β-HSD1, showing a significant positive correlation between BMI and 11β-HSD1 expression in different groups of subjects (CS and obese patients and control subjects).^87^

The lack of a correlation between circulating F levels and 11β-HSD1 expression in both obese subjects and Cushing's patients demonstrates that this enzyme is not directly regulated by plasma F concentrations. Surprisingly, we found 11β-HSD1 levels in visceral adipose tissue of patients with CS that are comparable to those observed in normal-weight control patients, which suggests that downregulation of the enzyme occurs as a result of long-term overstimulation.

Overall, data in the published literature indicate that 11β-HSD1 expression and activity are finely regulated tissue, specifically and strictly associated with energy balance status. This explains why 11β-HSD1 has been targeted therapeutically via the development of potent and specific inhibitors, including arylsulfonamidothiazoles,^88^ adamantly triazoles,^89^ anilinothiazolones,^90^ and as further demonstrated in phase II studies recently published (inhibitor INCB13739) showing an improvement of glycemic control in DM2 patients, together with a modest reduction in body weight.^91^ In adipose tissue, 11β-HSD1 inhibition is able to decrease lipolysis 92 and enhance lipogenesis, suggesting that the improvement of metabolic phenotype is likely mediated by decreased lipid mobilization.^83^

### Role of AMP-activated protein kinase

AMP-activated protein kinase (AMPK) is a key element of energy metabolism, implicated in the regulation of several metabolic pathways resulting in the inhibition of anabolic pathways (fatty acid, triglycerides, cholesterol, and protein synthesis) and activation of catabolic pathways (glycolysis and fatty oxidation). AMPK switches off ATP-consuming processes and switches on catabolic processes that produce ATP, thus restoring the AMP:ATP ratio. AMPK phosphorylates and inactivates two rate-limiting enzymes in fatty acid and cholesterol synthesis, acetyl-CoA carboxylase (ACC) and HMG-CoA reductase.^93^,^94^ Besides its lipid-related effects, AMPK has been implicated in carbohydrate and protein metabolism, cell-cycle regulation, and mitochondrisl biogenesis.

**Figure 4:**
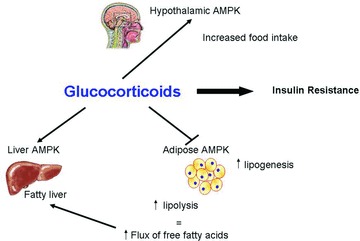
Summary of AMPK-mediated effects on different tissues and organs. AMPK switches off anabolic pathways and switches on catabolic pathways under the control of various metabolic hormones. Many metabolic effects exerted by glucocorticoids seem to be mediated by AMPK activation. In the central nervous system, AMPK activation favors increased appetite, while in the liver it leads to the development of hepatic steatosis through inhibition of gluconeogenesis and facilitation of lipid oxidation, associated with increased FFA availability due to AMPK inhibition in fat tissue, with consequent increased lipolysis and lipogenesis.

AMPK has a pivotal role in hypothalamic control of feeding behavior and is regulated by a wide variety of metabolic hormones, such as leptin, adiponectin, resistin, and ghrelin, and cannabinoids;^95^ it also acts in peripheral tissues, such as skeletal muscle and liver, where it regulates glucose metabolism and decreases glycogen synthesis and gluconeogenesis. Decreased AMPK activity in visceral fat tissue can stimulste lipolysis and lipogenesis, although the effect on lipogenesis is predominant (Fig. 4).^96^,^97^

It has been proposed that many of the negative effects exerted by GCs, especially those related to the development of metabolic alterations, could be mediated by GC-induced changes on AMPK activity, either directly or indirectly via stimulation of endocannabinoid synthesis. GCs can activate hypothalamic AMPK, which leads to a stimulation of appetite.^98^ In adipose tissue, GC treatment reduces AMPK activity rather than AMPK expression, which leads to increased lipogenesis and fat storage.^83^,^98^ GC's effects on adipocyte differentiation and lipid accumulation are more evident in visceral than in subcutaneous adipose tissue, which explains the development of central obesity observed during chronic GC exposure. Conversely, GCs were shown to increase AMPK activity in the rat liver, both *in vivo* and *in vitro*,^98^,^99^ and, consequently, due to suppression of gluconeogenesis and facilitation of lipid oxidation with increased production of FFA, development of hepatic steatosis is observed. Increased circulating levels of FFAs facilitate the development of insulin resistance and concomitant impairment of insulin signaling via reduced content and phosphorylation of insulin receptor substrate 1 (IRS-1).^100^–^102^ Finally, in the rat heart, GCs significantly decrease AMPK activity, suggesting that the observed detrimental effects of GC excess on the heart (i.e., left ventricular hypertrophy and myocardial ischemia), as is observed in patients with chronic exposure to excess GCs, could be, at least in part, mediated by the decrease in AMPK activity.^98^

In summary, several features that characterize GC's excess exposure—as occurs in CS patients—can be ascribed to AMPK activity modification. A tissue-specific modulation of AMPK activity seems to be responsible for the metabolic alterations observed in patients with CS, including visceral obesity, insulin resistance, glucose metabolism, lipid profile alterations, hepatic steatosis, and cardiac changes, which together characterize cardiometabolic syndrome.

## Adipose tissue and the renin–angiotensin–aldosterone system

**Figure 5:**
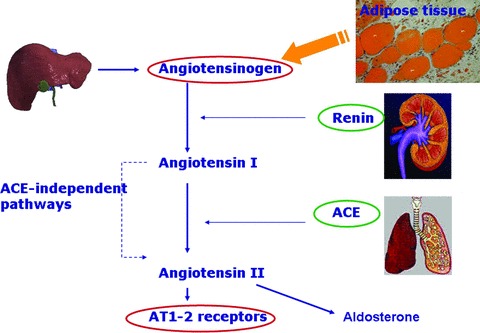
Schematic representation of the renin–angiotensin–aldosterone system (RAAS). Angiotensinogen (AGT) is secreted the liver and other tissues, such as adipose tissue. It is then converted by renin in angiotenin I, which is then transformed by the angiotensin-converting enzyme (ACE) in angiotensin II that, in turn, leads to adrenal production of aldosterone and has systemic effects on the cardiovascular system through its binding to AT1 and AT2 receptors.

Two distinct forms of the RAAS exist: a systemic or circulating form, and a local form that acts in peripheral tissues. Adipose tissue, especially the visceral type, possesses a local RAAS, which has paracrine as well as endocrine effects (Fig. 5).^103^ In the condition of visceral obesity, both local and systemic RAAS activity are increased.^103^ Such RAAS hyperactivity is responsible for the inhibition of preadipocyte differentiation and for the development of big insulin-resistant adipocytes able to secrete inflammatory adipokines, thus contributing to the cardiometabolic alterations associated with insulin resistance and hyperinsulinemia.^104^–^105^ In addition, in the presence of insulin resistance, it has been shown that increased FFA, through their effects on hepatic production of angiotensinogen (AGT), may stimulate aldosterone production, independent of renin.^103^

Pharmacological RAAS blockade seems to play an important positive role in insulin sensitivity. Numerous clinical trials have indicated that ACE inhibitors and angiotensin receptor blockers (ARBs) decrease the propensity to develop type 2 diabetes in high-risk patients,^106^–^108^ likely due to insulin sensitivity improvement.^109^

These effects have been explained, in part, by experiments in animal models with obesity and diabetes mellitus type 2. Treatment with different ARBs (olmesartan, valsartan, telmisartan, or candesartan) has been shown to induce a significant reduction in adipocyte size,^110^,^111^ body weight, and fat mass,^112^ all associated with improvements in insulin sensitivity, partly due to increased release of insulin-sensitizing adipokines and concomitant reduction of diabetogenic adipokines.^112^ In this regard, according to the hypothesis by Sharma *et al.*, while Ang II would inhibit preadipocyte differentiation, resulting in the formation of large insulin-resistant adipocytes,^113^ RAS blockade would induce preadipocyte recruitment, thereby increasing the number of small insulin-sensitive adipocytes that secrete fewer inflammatory cytokines and more beneficial cytokines, including adiponectin. The effects of ARBs in adipocyte differentiation seem to be mediated by an AT1R-blocking effect and, at least in part, by AT2R stimulation.

### RAAS and adipogenesis

It has been reported that some components of the RAAS (angiotensin II, angiotensin-converting enzyme (ACE), and plasma renin activity (PRA)) correlate with BMI.^105^ Indeed, increased circulating AGT, renin, aldosterone, and ACE activity have been described in obese patients.^114^,^115^ Moreover, increased RAAS gene expression has been found in adipose tissue of both obese animal models and obese humans.^116^–^118^

In animals, a trophic role for RAS on adipogenesis has been demonstrated using transgenic mice: the overexpression of AGT in adipose tissue is associated with increased fat mass, compared with wild-type mice, while AGT knockout mice present with reduced fat mass.^116^ In humans, increasing AGT, ACE, and renin mRNA expression has been observed during preadipocyte differentiation,^119^ thus indicating the relevance of local RAS on adipogenesis.

In addition, since the first studies on adipogenesis, which showed the ability of aldosterone to induce adipocyte differentiation,^120^ further steps forward have been achieved, first with the identification of the mineralococrticoid receptor (MR) expression in both in BAT and WAT,^121^,^122^ and then with the demonstration that such receptors are activated by aldosterone during adipose cell differentiation.^114^ Finally, more recently, it has been shown that aldosterone is able to induce adipose conversion of mouse cell lines in a time- and dose-dependent manner, and that MR, but not GR, knockdown inhibits the glucocorticoid-induced adipose conversion of 3T3-L1 cells.^123^

In subsequent studies, the same group has shown that selective blockade of the MR exerts antiadipogenic effects through an alteration of transcriptional control, which suggests a novel therapeutic option for fat deposition and its related metabolic sequelae.^124^

### Adipose tissue and aldosterone

A positive correlation between serum aldosterone levels and fat mass has been reported—especially in women—by several studies.^125^–^127^ Aldosterone levels not only positively correlate with visceral obesity^125^ and BMI^128^ but also fall in serum after weight loss.^127^ The mechanism by which weight loss can induce a reduction of aldosterone is not clear, although a reduced production of mineralocorticoid-releasing factors secreted by adipocytes^129^ could be involved. Besides genetic factors, additional factors have been proposed to explain the positive correlation between serum aldosterone levels and fat mass, for example, the involvement of adipose tissue RAS,^130^ the overactivity of the renal sympathetic nervous system, and stimulation of aldosterone secretion by an oxidized derivative of linoleic acid^131^ or by unknown potent fat mineralocortcoid releasing factors.^126^

Fat cell–conditioned media collected from primary cultures of human adipocytes have been shown to stimulate aldosterone secretion from NCI-H295R adrenocortical cells and from primary human adrenocortical cells (from normal adrenals) in an Ang II–independent way.^129^ Filtration of fat-conditioned media revealed the presence of two different fractions: an active one, with a molecular mass >50 kDa and representing 60% of the activity, and an inactive fraction with a mass of <3 kDa. Although the active molecules are still unidentified, the ability of adipocytes to secrete potent mineralocorticoid-releasing factors has been subsequently confirmed in a rat model of metabolic syndrome (SHR-cp).^132^ Once again, aldosterone-releasing activity of fat cell–conditioned medium was not angiotensin II mediated, as it was not inhibited by ARB candesartan. In addition, the authors showed that treatment of adrenocortical cells with fat-derived medium upregulated mRNA expression of StAR protein. The production of such fat-derived mineralocorticoid-releasing substances could contribute to the hyperaldosteronism observed in obese subjects.

## Aldosterone excess and glucose derangements in the clinical setting

The strict relationship linking aldosterone to adipose tissue has been underlined by several clinical and experimental studies conducted during the last few years. The deleterious effects exerted by aldosterone, via genomic and nongenomic actions in the heart, blood vessels, kidney, and brain have been extensively demonstrated both in experimental models and in humans.^133^ Less is known about the negative effects of such a hormone on glucose metabolism and insulin sensitivity, although some recent papers have underlined the relevance of aldosterone and its excess on the development of glucose metabolism alterations.^134^,^135^ In addition, a higher prevalence of glycemic abnormalities and of the metabolic syndrome has been demonstrated in patients with primary aldosteronism (PA), compared with patients with essential hypertension (EH).^136^,^137^

Conn *et al.*^138^ first reported an increased incidence of impaired glucose tolerance in patients with primary aldosteronism. In subsequent studies on the potential mechanisms involved in this impaired glucose intolerance, both consistent and conflicting data have been generated. Decreased insulin-receptor expression and affinity in subcutaneous adipose tissue of a patient with primary aldosteronism have been reported.^139^ Impaired pancreatic insulin has also been reported,^140^ and other reports^141^,^142^ have confirmed the finding of insulin resistance in primary aldosteronism patients.

As mentioned above, aldosterone levels positively correlate with visceral obesity and inversely with insulin sensitivity.^125^,^143^ A dose-dependent increase of aldosterone caused a decrease in glucose uptake together with increased expression of proinflammatory cytokines such as leptin and MCP-1, but this study was carried out in mouse brown adipocytes.^144^

The prevalence of insulin resistance in primary aldosteronism—compared with essential hypertension—and its response to treatment are also areas in which different findings have been reported. In a small group of patients, Sindelka *et al.* found reduced insulin sensitivity in those with primary aldosteronism compared with healthy controls. In addition, they reported significant improvement of insulin sensitivity after adrenalectomy but not after medical treatment of bilateral adrenal hyperplasia. First Widimsky, and then other authors, similarly found primary aldosteronism patients to be insulin resistant;^141^,^142^,^145^ on the other hand, they found no differences between patients with either primary aldosteronism or essential hypertension with regard to the prevalence of impaired glucose tolerance or diabetes.^146^ The study by Catena *et al.*^142^ produced findings different from those cited above in several ways. First, they found that although primary aldosteronism patients (with adrenal adenoma or bilateral adrenal hyperplasia) were more insulin resistant than age-, sex- and BMI–matched normal controls, the severity of the insulin resistance was less evident than in patients with EH. In addition, differently from our study, Catena *et al.* found an improvement in insulin sensitivity after surgical and medical treatment in patients with aldosterone-producing adenoma (APA) and those with idiopathic aldosteronism, respectively. Indeed, we have reported^147^ a distinction between treatment results in the two groups; on the one hand, surgical treatment of adrenal adenoma improved glucose tolerance evaluated by the 2-h oral glucose tolerance test, despite the increase of BMI, while on the other hand medical treatment in patients with idiopathic aldosteronism blocked further progression of the metabolic complications rather than reversed them. An increased rate of diabetes mellitus in primary aldosteronism was also found in patients in the German Conn's Registry, having PA patients compared with EH with a prevalence of diabetes of 23% versus 10%.^148^

Although the exact relationship among aldosterone, glucose metabolism, insulin action, and development of multiple sclerosis remains mostly unresolved, several factors have been proposed as pathogenetic elements, including an indirect effect via hypokalemia, which seems responsible for a reduction in insulin secretion—even though the precise role of this ion deficiency remains unclear, and in the light of the persistence of insulin resistance in PA patients during potassium infusion.^149^ In a previous study we showed that in patients with PA, homeostasis model assessment–estimated insulin resistance (HOMA IR) is higher in hypokalemic patients, thus indicating a possible effect of potassium on insulin sensitivity. It has also been suggested that nongenomic actions of aldosterone might result in an increase of collagen synthesis and fibrosis, not only in the heart but also in other tissues, such as the pancreas, liver, fat, and muscle, resulting in alterations of insulin release and insulin sensitivity.^150^

For pathogenetic mechanisms, great attention has been paid to adipose tissue and to the several recently discovered adipokines involved in glucose homeostasis. According to different studies, patients with PA present suppressed leptin levels,^151^,^152^ reduced adiponectin levels,^153^ and increased values of resistin.^154^ In this light, adipose tissue seems to play a role in the pathogenesis of glucose metabolism alterations in PA patients.^155^ To support this hypothesis, experimental studies with rodents have shown that aldosterone is able to increase the expression of proinflammatory adipokines responsible for a reduction in insulin receptor expression and impaired insulin-dependent glucose uptake.^156^ However, it has been recently demonstrated that gene expression of insulin signaling/inflammatory molecules (PPAR-γ, insulin receptor, GLUT-4, IRS-1 and 2, leptin, adiponectin, IL-6, MCP-1, 11βHSD1, 11βHSD2, and GR) was similar in visceral adipose tissue of patients with APA compared with adipose tissue of patients with a nonfunctioning adrenal adenoma. These data do not support an effect of aldosterone excess on adipose tissue–mediated insulin sensitivity.^157^

Nevertheless, our unpublished data regarding mRNA expression studies on visceral adipose tissue samples of APA patients, compared with samples of healthy controls and patients with a nonfunctioning adrenal adenoma, showed significantly higher expression, in samples of APA patients, of IL-6, an important adipokine with proinflammatory properties and a marker of insulin resistance. The association of IL-6 and insulin resistance seems complex, and evidence suggests that IL-6 might act at multiple levels, both centrally and on peripheral tissues, to influence body weight, energy homeostasis, and insulin sensitivity.^158^

Finally, recent evidence indicates that the detrimental effects of aldosterone excess on insulin signaling are mediated by inflammatory/oxidative stress mechanisms of mineralocorticoids.^159^ To support the involvement of the aldosterone stress mechanism in the TG(mRen2)22 rat, which has insulin resistance,^160^*in vivo* MR antagonism with spironolactone substantially improves *ex vivo* insulin-stimulated increases in glucose uptake in skeletal muscle.

## Conclusions

A tight link between adipose tissue and the adrenal gland has been widely demonstrated. From a physiological point, GCs are essential for adipocyte biology, in particular for differentiation and lipid homeostasis.^80^ From a clinical point of view, the dysregulation of both the hypothalamic–pituitary–adrenal axis (HPA) and of cortisol metabolism are implicated in the development of visceral obesity, metabolic syndrome, and cardiovascular diseases.^41^ Such characteristics are indeed typically observed in the presence of hypercortisolism, both endogenous, that is, CS, as well as in exogenous hypercortisolism. Similarly, the mineralocorticoid counterpart also has a role in adipocyte differentiation^120^ and seems to be, at least in part, regulated by adipose-secreted factors, with reciprocal effects between adrenal gland and adipose organ.^126^ Such interactions, in the presence of pathological condition, such as primary aldosteronism, result in the development of metabolic alterations. The picture is complicated by the interplay between cortisol and aldosterone and their respective receptors. It is indeed well known that the GR and MR display both structural and functional homology. The fact that cortisol is able to bind to the MR with an affinity higher than the affinity of aldosterone itself explains why it has been proposed that in the presence of chronic cortsiol excess this hormone could act via MR activation. In the same way, aldosterone action in particular conditions is mediated through GR activation. Such interplay is particularly evident in adipose tissue, and on adipogenesis and adipose biology.
